# *Scutellaria baicalensis* and Their Natural Flavone Compounds as Potential Medicinal Drugs for the Treatment of Nicotine-Induced Non-Small-Cell Lung Cancer and Asthma

**DOI:** 10.3390/ijerph18105243

**Published:** 2021-05-14

**Authors:** Naser A. Alsharairi

**Affiliations:** Heart, Mind & Body Research Group, Griffith University, Gold Coast, QLD 4222, Australia; naser.alsharairi@gmail.com

**Keywords:** *Scutellaria baicalensis*, flavone compounds, nicotine, NSCLC, asthma

## Abstract

Flavonoids as the largest group of natural phytochemical compounds have received significant attention, as demonstrated by clinical trials, due to their chemotherapeutic and/or pharmacological effects against non-small-cell lung cancer (NSCLC) and asthma. *Scutellaria baicalensis* (*S. baicalensis*), known as one of the most popular medicinal plants and used in several countries, contains natural active flavone constituents, with the major compounds of the roots being baicalein, baicalin, wogonin, wogonoside and oroxylin A. *S. baicalensis* and their compounds are proven to have inhibitory effects on NSCLC cells when used at different concentrations. However, the exact mechanisms by which these compounds exert their therapeutic effects against asthma remain unexplored. Indeed, the mechanisms by which *S. baicalensis* and its flavone compounds exert a protective effect against nicotine-induced NSCLC and asthma are not yet fully understood. Therefore, this review explores the mechanisms involved in the therapeutic potential of flavone-rich extracts from *S. baicalensis* in nicotine-induced NSCLC and asthma.

## 1. Introduction

Lung cancer is regarded as the most common cause of cancer-related mortality worldwide [1] and is mostly caused by malignant tumors [2]. Lung cancer consists of two main types: small cell lung cancer (SCLC) and non-small-cell lung cancer (NSCLC), with the latter comprising 85% of all lung cancer cases, and including three smoking-related subtypes; squamous, adenocarcinoma and large cell carcinoma [3]. Globally, more than 64% of lung cancer mortality is attributed to tobacco smoking [4]. Nicotine as the major addictive and toxic chemical ingredient in cigarette smoke increases risk of developing NSCLC [5,6,7]. Nicotine and cigarette smoke extract (CSE) are involved in inducing Epithelial-to-Mesenchymal Transition (EMT) through epigenetic change and histone deacetylase (HDAC)-mediated downregulation of epithelial (E)-cadherin and increasing expression of the mesenchymal markers metalloproteinase 9 (MMP9), vimentin, fibronectin and neural (N)-cadherin in NSCLC cell line A549, leading to lose adhesions/junctions and polarity in epithelial cells [8,9,10].

In NSCLC, tumor cell interaction with immune system is controlled by a network of biological signaling pathways [11,12]. T-cells responses have been observed against NSCLC tumor-associated antigens (TAA) (e.g., MAGE-A1, PRAME, MUC1) in NSCLC cell lines [13], which may result in activation of inhibitory immune checkpoint pathways/molecules such as programmed death-1 (PD-1), programmed death-ligand 1 (PD-L1) and cytotoxic T-lymphocyte-associated protein 4 (CTLA-4), in what are known as markers for exhausted T cells [12]. Upon the activation of antigen, the histocompatibility complex (MHC) class I and II molecules on antigen-presenting cell (APC) present tumor antigens to the T-cell receptor (TCR), resulting in an activation of TAA-specific CD8+ cytotoxic T cells and CD4+ helper T cells. Inhibitory immune checkpoint pathways are expressed on regulatory T cells (T_regs_) after their upregulation, and function as immunosuppressive of CD8+ and CD4+ T cells via further production of pro-inflammatory cytokines, including interleukin (IL)-10 and transforming growth factor β (TGFβ) [12].

Asthma is a chronic inflammatory disease in which airway smooth muscle (ASM) contributes to asthma through airway wall thickening, hyperresponsiveness and remodeling, impaired relaxation and persistent airflow obstruction [14]. There have been studies indicating that smoking is a potential risk factor for asthma, and the risk of asthma appears to increase among smokers [15]. Nicotine is thought to play a key role in the development of asthma in adult smokers [16]. Asthma is regarded as a contributing factor in lung cancer, and the risk of NSCLC is increased greatly in asthmatic smokers [17].

Several systemic treatments for inoperable NSCLC exist including, chemotherapy, immunotherapy and molecular targeted therapies, while surgery and radiation are used in patients with locally advanced [18]. NSCLC is still the leading cause of death, despite the prominent progress in the prognosis over the last years [1]. Monoclonal antibodies such as atezolizumab, nivolumab, pembrolizumab and bevacizumab targeting inhibitory immune checkpoint molecules have shown novel immunotherapies agent in locally advanced NSCLC [11,19]. Treatment with chemotherapeutic agents (e.g., cisplatin) in combination with monoclonal antibodies may provide benefits in patients with metastasized stage NSCLC [19,20]. Therapeutic agents such as afatinib, osimertinib, gefitinib and erlotinib have shown promising results in patients with NSCLC who have driver mutations such as epidermal growth factor receptor (EGFR) and anaplastic lymphoma kinase (ALK) [19]. Pycnogenol and quercetin have been widely used in asthma treatment, since they are found to suppress inflammatory cytokine production and airway hyperresponsiveness [21]. With several dietary supplements available in a global market, there is still a research gap on their efficacy and safety to treat lung cancer, particularly among asthmatic smokers, given inconsistent results in clinical trials, with no benefits, and in fact, it has been shown that they may cause harm in long-term use [21,22]. Therefore, promising therapeutic agents with low toxicity and high efficacy in targeting NSCLC and asthma in patients are needed.

Flavonoids are the major group of natural plant-derived compounds with polyphenolic structure, and are largely present in grains, flowers, roots, seeds, fruits, vegetables and drinks (including wine, cocoa and tea) [23]. Flavonoids are categorized into distinct classes depending on their chemical structure, with a fifteen-carbon skeleton comprising of two aromatic benzene rings (A and B) and connected by a third heterocyclic pyrane ring (C) [24]. Major classes include isoflavones (e.g., genistein, genistin, glycitein, daidzein, daidzin), flavanonol (e.g., taxifolin), flavanones (e.g., naringenin, naringin, hesperidin, hesperitin, abyssinones, eriodictyol), flavonols (e.g., galangin, quercetin, myricetin, rutin, morin, fisetin, kaempferol), flavones (e.g., chrysin, diosmetin, tricin, luteolin, apigenin, baicalin, baicalein, tangeretin, rpoifolin), flavan-3-ols (e.g., epicatechin gallate, catechin), anthocyanidins (e.g., petunidin, pelargonidin, delphinidin, peonidin, malvidin) and chalcones (e.g., phlioridzin, chalconaringenin, phloretin, arbutin) [23,24]. The substitution and/or configuration patterns of the hydroxyl groups in the flavonoid classes A, B- or C-rings may impact their biological activities, particularly in terms of antioxidant, antibacterial, antiviral and anti-inflammatory properties and modulation of major enzymatic pathways, which are considered key mechanisms through which these classes could exert chemopreventive effects against cancer [23,24,25]. A recent review showed that flavones, and apigenin, diosmetin and luteolin in particular, act as anti-lung cancer drugs, which have a wide range of molecular mechanisms for the prevention and/or treatment of NSCLC cells. The main mechanisms include (1) suppression of receptor tyrosine kinase (RTK), which is able to mediate activation of cellular signaling pathways, leading to the promotion of lung cancer tumor growth, (2) suppression of proliferation in lung cancer cells, (3) induction of apoptosis and cell cycle arrest through activation of protein 53 (p53) expression and inhibition of key transcription factors, and (4) attenuation of the invasion/migration and metastasis of NSCLC cells through inhibition of the EMT [26]. A recent study on NSCLC cell line A549 after treatment with luteolin demonstrated significant suppression of the migration/invasion and filopodia formation, with no cytotoxic activity in cells observed. The effect of luteolin is induced via down-regulating expression of the focal adhesion kinase (FAK) and non-receptor tyrosine kinase (Src) downstream pathways, including the Ras homolog gene family member A (RhoA), cell division control protein 42 (Cdc42) and Ras-related C3 botulinum toxin substrate 1 (Rac1) [27].The natural flavone luteolin has been reported to suppress mast cells (MCs) that mature in human tissues and are implicated in asthma. Luteolin is significantly suppressed immunoglobulin E (IgE)/anti-IgE-induced histamine, beta-hexosaminidase (β-hex) and tumor necrosis factor (TNF)-α production from laboratory of allergic diseases 2 (LAD2) human MCs. The potential mechanisms underlying the anti-inflammatory action of luteolin involve inhibiting nuclear transcription factor-kappaB (NF-κB) activation and intracellular calcium increase [28].

There has been an increased interest in ascertaining the significance of medicinal plants in the treatment of diseases, given the high content of flavonoid compounds in these plants [24]. Medicinal plants have proven clinical efficacy for the treatment of a plethora of diseases such as cardiovascular disease [29], neurodegenerative disorders [30] and rheumatoid arthritis [31]. Medicinal plants have also been proven to be effective in asthma [32] and NSCLC treatment [33]. However, a significant number of medicinal plants can produce toxic side effects and their safety for use by patients is uncertain [34,35,36]. Therefore, further studies are needed to find potent medicinal plants useful for NSCLC and asthma treatment with few or no side effects.

*Scutellaria baicalensis* Georgi (*S. baicalensis*), also known as Chinese skullcap orHuang-Qin, is a traditional Chinese medicinal plant that belongs to the family Lamiaceae and is widely used in East Asian countries, Russia, Europe and North America as an adjuvant to chemotherapy for many diseases [37,38,39]. Flavonoids and their glycosides are the major bioactive chemical compounds of *S. baicalensis*, with the main constituents of the root-specific 4′-deoxyflavones (known as *Scutellariae radix*, *S. radix*) being baicalein, baicalin, wogonin, wogonoside and oroxylin A [38,39]. These flavones exert anti-tumor functions against various types of cancer, including brain, prostate and oral squamous cell carcinoma (SCC) [40], and have been recorded in Chinese and European Pharmacopoeia as being medicinal drugs used to treat several diseases [39]. Previous studies are still insufficient to understand the effect of flavone compounds in NSCLC treatment in vivo. Studies in mice showed that A549 and H-460 cells treated with *S. baicalensis* compounds (baicalin, baicalein and wogonin) result in inhibition of cell proliferation and angiogenesis by downregulating expressions of inhibitors of differentiation 1 (Id1) protein, EMT-related molecules (N-cadherin, vimentin), vascular endothelial growth factor A (VEGF-A) and fibroblast growth factor receptor 2 (FGFR2) through suppression of the Src/Id1 signaling pathway [41,42,43]. The molecular effect and mechanism of action of *S. baicalensis* and its compounds at different concentrations showed strong inhibitory properties in NSCLC cells, as reported by in vitro studies. However, the potential molecular mechanisms by which *S. baicalensis* and its isolated compounds are thought to be useful in the treatment of asthma have not been clearly studied. Indeed, the mechanisms of the anti-lung cancer and asthma action of *S. baicalensis* containing flavones compounds in nicotine-induced NSCLC and asthma are unclear. The significant link between NSCLC and asthma could have health benefits for smokers in terms of using *S. baicalensis* and its compounds. Given that *S. baicalensis* is used in several therapeutic practices, targeting the flavones extracted from such a plant and understanding its molecular mechanisms of action might help in providing new therapeutic strategies for the treatment of NSCLC and asthma, particularly in smoker patients. To date, there are no reviews which discuss the mechanisms of *S. baicalensis* and their compounds involved in nicotine-induced NSCLC and asthma treatment. Therefore, this review attempts to provide a detailed overview of the mechanisms of flavones extracted from *S. baicalensis* and explore its therapeutic potential in nicotine-induced NSCLC and asthma.

## 2. Methods

A literature search was undertaken to retrieve studies published in English and carried out over the last 10 years, using the PubMed/MEDLINE database and the following search words: “*Scutellaria baicalensis*” or “*Scutellariae radix*” or “baicalein” or “baicalin” or “wogonin” or “wogonoside” or “oroxylin A” and “lung cancer” or “NSCLC” and “asthma” and “smoking” or “nicotine”. Studies on *S. baicalensis*/*S. radix* and their compounds are included if they have been tested for anti-NSCLC and asthma efficacy. The search is limited to experimental in vitro studies. In total, 216 articles were extracted using the search terms. As a result, 29 studies were selected based on the search criteria.

## 3. In Vitro Effects of Nicotine on NSCLC Development

Nicotine as the main addictive and harmful chemical constituent of cigarette smoke plays a key role in lung carcinogenesis [5,6,7], which enhances proliferation, invasion, cell cycle progression, angiogenesis, and inhibition apoptosis through activation of the a7 subunit of the nicotine acetylcholine receptor (a7nAChR)-mediated signaling pathways implicated in NSCLC development [44,45,46]. Activation of nicotine-α7nAChR promotes cell invasion/proliferation in NSCLC cells, and EMT which is a key step in altering the malignant phenotype of cancer cell lines, resulting in suppression of E-cadherin and induction of MMPs, vimentin, fibronectin and N-cadherin expression [8,9,10]. Upregulation of several transcription and growth factors such as TGFβ, Snail, twist, deca-pentaplegic homolog (Smad) and cyclinD1, as well as hypoxia-inducible factors (e.g., HIF-1α) induce EMT in NSCLC cells via activation of the Notch signaling pathway [47,48]. The α7nAChR expression level has been revealed to be higher in SCC compared to other NSCLC types, particularly in smokers [46]. Nicotine-specific nitrosamines, including N′-nitrosonornicotine (NNN) and 4-(metylnitrosamino)-1-(3-pyridyl)-1-butanon (NNK) have the most carcinogens in cigarette smoke, which in turn after binding to α7nAChR and nicotinic acetylcholine receptors (β-AR) (e.g., adrenaline and noradrenaline) activate the EGFR/serine-threonine kinase (EGFR/Akt) signaling pathway involved in anti-apoptosis, which contributes to NSCLC progression in smokers [49]. The nicotine-mediated activation of α7nAChR and its downstream signaling pathways are involved in several functions, as reported by in vitro studies [46,49,50]. Table 1 shows the functions of nicotine/α7nAChR-mediated NSCLC development.

## 4. Therapeutic Role of *Scutellaria baicalensis* and Their Flavone Compounds in Nicotine-Induced NSCLC

This section presents the therapeutic role of *S. baicalensis* and their flavone compounds in nicotine-induced NSCLC.

*S. baicalensis* and its extracts possess anti-lung cancer activity demonstrated in NSCLC cells. Park et al. [51] showed that the aqueous extract of *S. baicalensis* root inhibits cell motility/proliferation and induces G1 phase arrest, with no cytotoxicity against A549 cells observed. *S. baicalensis* downregulates cyclinD1, cyclin-dependent kinase 4 (CDK4) and MMP2 production in A549 cells. Kim et al. [52] reported that the aqueous extract of *S. radix* induces apoptosis by increasing caspase 3, Poly ADP ribose polymerase (PARP) cleavage, and phospho-AMP-activated protein kinase (AMPK) and reducing mTOR in H2087 and H358 cells. *S. radix* also induces autophagy via increasing microtubule-associated protein 1A/1B-light chain 3 (LC3)-II/LC3-I expression. A study by Wang et al. [53] assessed the anti-lung tumor effect of *S. radix* ethanolic extracts on cell invasion and proliferation of A549 cells. The extracts (baicalein, wogonin) cause invasion and proliferation inhibitory effects on A549 cells and arrest the cell cycle at S phase via downregulating expression of cyclinD1 and upregulating expression of p53.

Gao et al. [54] studied the anti-lung cancer effect of *S. baicalensis* ethanolic extracts (baicalein, baicalin, wogonin) on NSCLC cells. Cytotoxicity analysis has demonstrated strong activity against cell lines SK-LU-1, A549 and SK-MES-1. The cytotoxicity of *S. baicalensis* is reported as high against A549 and SK-LU-1 cells due to the stochiometric combination of extracts. The extracts reported cell cycle and apoptosis-inducing in NSCLC cells via upregulating expressions of p53 and Bax and downregulating expressions of cyclinA. The study concludes that the cytotoxicity of extracts may control growth of NSCLC cells via inducing cell cycle arrest and apoptosis, suggesting the significance of cell growth arrest and apoptosis as potential mechanisms in the cytotoxicity of flavones treatment. Gong et al. [55] studied the apoptotic and anti-inflammatory effects of baicalin, baicalein and wogonin extracted from *S. baicalensis* on nicotine-induced A549 and H1299 cells. The extracts upregulate expression of Bax and downregulate expressions of MMP2, MMP9, caspase-3, bcl-2/bax ratio and bcl-2. The extracts showed anti-inflammatory activity by inhibiting expressions of NF-κB, TNF-α, IL-6 and I kappa B-alpha (IκB-α) in NSCLC cells. Zhao et al. [41] reported the effects of three major *S. baicalensis* extracts: baicalein, baicalin and wogonin on A549 and H1299 cells. The extracts inhibit growth, and the invasive and migratory activities of NSCLC cells. The extracts suppress expressions of VEGF-A, N-cadherin and vimentin through Akt/Src signaling pathway blocking.

### 4.1. Baicalein

Baicalein has been reported to promote apoptosis and/or suppress cellular growth and proliferation of NSCLC cells. A study has shown that baicalein promotes sensitivity to cisplatin drugs in A549 and H460 cells by inducing apoptosis and inhibiting cell proliferation through suppression of the PI3K/Akt signaling pathway and the expression of microRNA 424-3p (miR-424-3p) targeting phosphatase and tensin homolog (PTEN) gene expression [56]. In another study, baicalein enhances antitumor activity by reducing the induction of vasculogenic mimicry (VM), a pattern of tumor microcirculation, via inhibiting the expression of VM-associated factors (e.g., MMP9, VE-cadherin) and the RhoA/rho-associated coiled-coil kinase (RhoA/ROCK) signaling pathway, leading to the suppression of motility and viability of A549 cells [57]. A study by Li et al. [58] reported that baicalein suppresses MAP4K3 expression via reducing its stability. The results also indicated that inhibition of the mTOR signaling pathway in A549 and H1299 cells by RNA interference could be sufficient in mediating baicalein-induced autophagy.

In a study carried out by Zhang et al. [59], the A549 and H1299 cells were treated with different concentrations of baicalein to study the mechanisms by which baicalein suppresses cell metastasis. The results showed that baicalein inhibits cell metastasis by inhibiting ezrin tension transduction, leader cell production and inducible nitric oxide synthase (iNOS)-mediated ezrin S-nitrosylation (SNO) tension, which are responsible for A549 and H1299 cell aggression in the inflammatory milieu. A study by Cathcart et al. [42] reported that the angiogenesis of NSCLC cells (A549, H460 and SKMES1) is significantly inhibited via downregulating VEGF-A, FGFR-2 and RB-1 expression. Su et al. [60] reported baicalein increases the protein expression of E-cadherin and inhibits the EMT-induced N-cadherin and vimentin in A549 and H1299 cells by downregulating the Notch signaling pathway. Similarly, a study on the anti-tumor effect of baicalein on NSCLC cells showed that baicalein causes an inhibitory effect on A549 cells by suppressing the expressions of N-cadherin, vimentin, VEGF-1, and upregulating the expression of E-cadherin through Src-Id1 signaling pathway blocking [43]. Therefore, baicalein showed effective results in the inhibition of NSCLC cells through anti-proliferative/metastatic, anti-angiogenesis and apoptosis/autophagy induction. The mechanisms underlying the effect are linked to the inhibition of different cellular signaling pathways.

### 4.2. Baicalin

A few in vitro studies have shown potent anti-lung cancer activity of baicalin in NSCLC cell lines. Zhang et al. [61] reported the anti-lung cancer activities of baicalin in A549 and H2009 cells at different concentrations, in combination with tumor necrosis factor-related apoptosis-inducing ligand (TRAIL), a potential anticancer agent which modulates signaling pathways in NSCLC cells. Baicalin sensitizes lung cancer cells to TRAIL-induced apoptosis via the activation of MAPK and reactive oxygen species (ROS) production. Another study reported a high anti-lung cancer effect of baicalin in combination with DDP (cisplatin) on proliferation and invasion of A549 cells. Baicalin suppresses protein expression of microtubule affinity-regulating kinase 2 (MARK2) and Akt in A549/DDP cells, which are involved in cell proliferation and the pathogenesis of lung cancer [62]. Diao et al. [63] used baicalin to examine its inhibitory effect on NSCLC cells. The inhibitory effect of baicalin is observed against H441, H1975 and H1299 cells with a high expression level of PDZ-binding kinase/T-LAK cell-originated protein kinase (PBK/TOPK), a novel mitotic kinase which promotes cell invasion. You et al. [64] reported that baicalin stimulates apoptosis and inhibits the invasion abilities in A549 and H1299 cells. This is observed through activation of the sirtuin 1 gene/AMPK (SIRT1/AMPK) signaling pathway, known as tumor suppressor genes, and inhibition of the expression levels of MMP2 and MMP9 in NSCLC cells. From these studies, it can be suggested that baicalin has significant anti-proliferative/invasive and apoptotic effects, and the mechanisms of its therapeutic effect in NSCLC cells are related to the modulation of cellular signaling pathways.

### 4.3. Wogonin

Wogonin possesses antitumor properties against NSCLC cells, as demonstrated by a few in vitro studies. The study conducted by He et al. [65] found high anti-tumor activity against A549 cells of wogonin in combination with cisplatin. The anti-tumor effect of wogonin is reported as enhancing cisplatin-induced cell death through activating apoptosis, cleavage of the caspase-3 substrate PARP, caspase-3 and ROS production. Zhao et al. [66] assessed the inhibitory effect of wogonin in A549 cells by targeting the inflammatory microenvironment. The results demonstrated inhibition in human monocytic leukemia cell line (THP-1 CM)-induced migration of A549 cells, IL-6-induced EMT, vimentin, N-cadherin, Twist and Snail, and activation of E-cadherin. The inactivation of signal transducer and activation of the transcription 3 (STAT3) signaling pathway was found to be the mechanisms for this effect.

Wang et al. [67] performed a proliferation inhibition assay on A549 cells. The study reported a significant cell proliferation inhibition rate of 54% in A549 when treated with wogonin. Wogonin also showed an effect on energy metabolism by reducing lactate dehydrogenase (LDH) activity in A549 cells. In a study performed to assess the effect of wogonin on NSCLC cells and to explore their mechanism of action as an anti-tumor inhibitor, wogonin showed a significant cell viability inhibition rate of 31% and 34% in A549 and A427 cells, respectively. The mechanism of NSCLC cell inhibition is linked to the activation of apoptosis-inducing enzymes, autophagy and formation of ROS [68]. In the study by Chen et al. [69], the anticancer effects of wogonin in different concentrations on A549 cells are examined through regulating cellular pathways to assess cell viability. Results showed that wogonin inhibits cell viability and induces apoptosis in A549 cells through down-regulating HDAC1 and HDAC2 at mRNA and protein levels, and c-Myc oncogene, S-phase kinase-associated protein 2 (Skp2), F-box and WD repeat domain-containing 7 (Fbw7) and Glycogen synthase kinase 3b (GSK3b) at the protein level. A study of the inhibitory effect of wogonin on cell viability of NSCLC cells, Shi et al. [70] reported cell cycle arrest, senescence and apoptosis-induction in A549 cells by the downregulation of protein serum levels and glucocorticoid-inducible kinase 1 (SGK1). Furthermore, wogonin upregulates cellular levels of apoptotic protease activating factor-1 (APAF1), Bax, p21, promyelocytic leukemia protein (PML), growth arrest and DNA damage-inducible 45A(GADD45A) and yippee-like 3 (YPEL3) in A549 cells, which are the key factors involved in apoptosis, senescence and cell cycle arrest. Therefore, it can be suggested that wogonin has a strong anti-proliferative/viability, anti-inflammatory and apoptotic activities against NSCLC cells through regulation of several cellular signaling pathways as the mechanisms for these activities.

### 4.4. Wogonoside

Studies that have examined the in vitro activities of wogonoside on NSCLC cells are limited. A study by Luo et al. [71] reported the inhibition of A549 cells via inducing apoptosis and cell cycle arrest when treated with wogonoside. Wogonoside treatment upregulates cleaved caspase-3/9 and Bax expression, downregulates Bcl-2 expression, and promotes the levels of mitochondrial cytochrome *c* in the cytosol of A549 cells. Furthermore, wogonoside induces apoptosis and cell cycle arrest by reducing phospho-mTOR and its downstream target p70S6K and increasing phospho-AMPK in A549 cells, suggesting that the AMPK/mTOR signaling pathway may be involved in the apoptotic activity of wogonoside. A study by Wang et al. [72] did not show any anti-proliferative effects of wogonoside on six cancer cell lines, including NSCLC (DMEM). However, the study observed the anti-proliferative activity on DMEM treated with wogonin (the deglycosylation of wogonoside).

### 4.5. Oroxylin A

**Figure 1:**
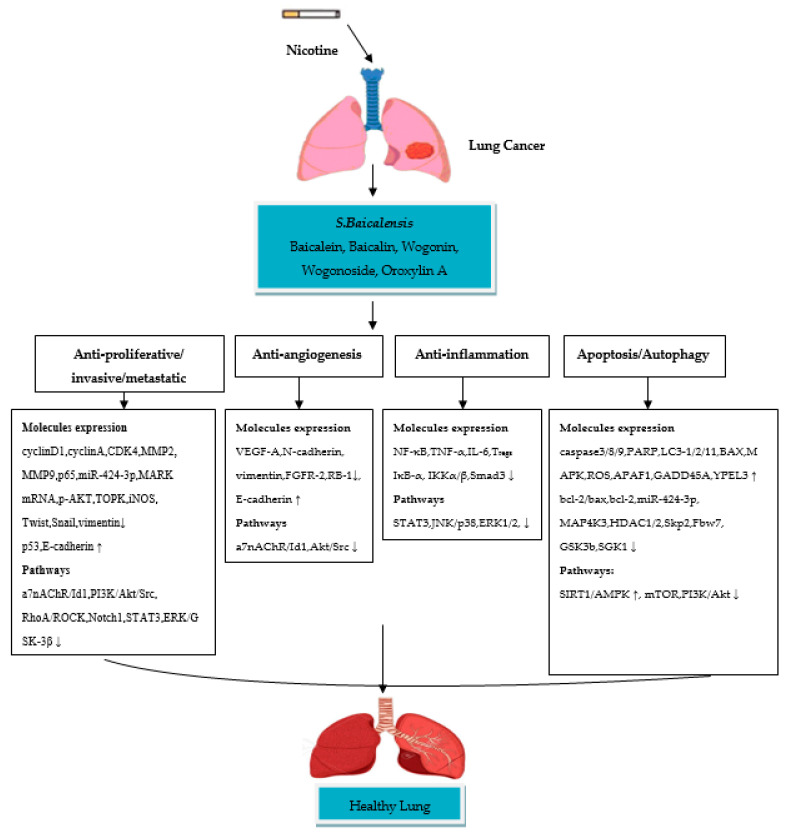
Schematic diagram of the molecular mechanisms of *S. baicalensis* compounds in nicotine-induced NSCLC treatment. (↓) decrease, (↑) increase.

A few in vitro studies have revealed that oroxylin A (OA) possesses strong anti-lung cancer activity in NSCLC cell lines. Shen et al. [73] studied the anti-inflammatory effect of OA on T_regs_ in the lung cancer environment by using the H460 cell line. The results of the study showed that OA inhibits T_regs_ production in H460 cells. Treatment with OA causes an inhibitory effect on the H460 cells cocultured with Jurkat cells by suppressing TGFβ-activated Smad3, ERK1/2, c-JUN NH2-terminal kinase (JNK) and p38 signaling pathways. OA also significantly inhibits the DNA binding activity of NF-κB/p65 in H460 cells by reducing phospho-Kappa B kinase α, β (IKKα, IKKβ). A study by Wei et al. [74] has been performed to assess the anti-invasive effect of OA on NSCLC cells and the molecular mechanisms involved. The study reported inhibition in A549 and 95-D cell migration/invasion with a 70% and 73% inhibition rate OA, respectively. The mechanism of inhibition is attributed to the suppression of Snail/TGFβ-induced EMT through ERK/Glycogen synthase kinase-3β (GSK-3β) signaling pathway blocking. As a result, OA downregulates expression of CD44v6, MMP-9, and vimentin and upregulates the expression of E-cadherin, and this could lead to the suppression of invasion and migration in Snail-expressing NSCLC cells. Another study by Wei et al. [75] reported the effect of OA on anoikis (cell death)-sensitization and glycolysis-inhibition in NSCLC cells. Results showed that OA suppresses the growth of detached A549 cells and induces anoikis through Src/PI3K/AKT signal pathway inhibition at the Tyr418 and ser316 sites. The study also showed that OA induces anoikis in A549 cells by lowering ATP synthesis, the level of glycolysis, lactic acid production and hexokinase II (HK II) glycolytic enzyme activity in mitochondria. A study by Liu et al. [76] was conducted to evaluate the effect of OA with cisplatin on suppression of NSCLC cells under hypoxic conditions. Flow cytometric and CCK8 assays reported that OA promotes cisplatin-induced cell death in H460 cells under hypoxia through inhibiting xeroderma pigmentosum group C (XPC), a DNA damage recognition protein upregulated by HIF-1α, which is implicated in the activation of nucleotide excision repair (NER). It is, therefore, suggested that OA has significant anti-invasive and anti-inflammatory activities in nicotine-induced NSCLC cells, due to their ability to inhibit cellular signaling pathways involved in NSCLC development. The in vitro studies on the anti-NSCLC activity of *S. baicalensis*/*S. radix* and their compounds, as well as their molecular mechanisms of action are summarized in Table 2. Taken together, *S. baicalensis* compounds possess anti-proliferative/metastatic/invasive, anti-inflammatory, anti-angiogenesis and apoptotic/autophagic properties (Figure 1). These compounds showed significant inhibition of NSCLC cells by suppressing/modulating the activity of cellular signaling pathways.

## 5. In Vitro Effects of Nicotine on Asthma Development

Nicotine has been shown to activate the α7 nAChR signaling pathway in lung fibroblasts to induce NFκB transcriptional activity/nuclear translocation and produce nerve growth factor (NGF) in smokers, which contributes to airway hyperresponsiveness [77]. Nicotine induces upregulation of human bronchial smooth muscle cells (HBSMCs) proliferation by increasing receptor-operated Ca^2+^ entry (ROCE), store-operated Ca^2+^ entry (SOCE) and basal [Ca^2+^]_i_ concentrations in these cells, which results through activation of the α7nAChR-mediated PI3K/Akt signaling pathway and upregulation of transient receptor potential protein 6 (TRPC6) mRNA and protein expression in HBSMCs [78]. The nicotine component of CSE contributes to asthma by promoting [Ca^2+^]_I_ responses of human airway smooth muscle (hASM) cells to agonist and SOCE through upregulation expression of Orai1, CD38, stromal interaction molecule 1 (STIM1) and TRPC3 in hASM cells, which are the key molecular players for activation of sarcoplasmic reticulum Ca^2+^ release from ryanodine receptor channels [79].

Nicotine promotes the release of pro-inflammatory cytokines such as IL-17A, IL-6 and IL-8 in the bronchial mucosa of asthmatic smokers [80]. IL-17A has the ability to stimulate TGF-β1, which contributes to abnormal epithelial barrier function, hASM contraction and airway smooth muscle remodeling in asthma and correlates with promoted TH17 activity in secreting proinflammatory cytokines through NF-κB, PI3K and MAPK signaling pathways activation [81]. IL-17A plays a key pathogenic role in asthma, as it induces some of the growth-related oncogene (GRO) chemokine production (e.g., CXCL2 and CXCL3) [82], which enhance asthmatic airway smooth muscle cell (ASMC) migration by inducing ERK1/2 and p38 MAPK signaling pathways activation via CXCR1 and CXCR2 chemokine receptors [83]. IL-17A and IL4 have a potential synergistic effect with TGF-β1 in inducing bronchial EMT, p-Smad3 expression and ERK1/2 signaling pathway activation, and inhibiting E-cadherin mRNA expression in the setting of severe asthma [84]. Research evidence points to an increased IL-4, Th17 and the levels of serum mTOR as playing a key role in asthma development. The mTOR pathway activation in asthmatic patients is demonstrated by Th1/Th2 imbalance and loss of Th17/T_reg_ [85]. Activation of the ROS/PI3K/Akt/mTOR pathway by CSE plays a key role in cell senescence in asthma [86,87] and may contribute to lung inflammation and alterations in asthma [88].

Nicotine has been shown to induce EMT via Wnt/β-catenin activation in hASM cells. This process is accompanied by an increase in MMP9, type I collagen and vimentin expression, as well as inhibition of E-cadherin [89]. Nicotine upregulates cyclinD1 expression in HBSMCs in smokers with asthma [90] and also promotes hASM proliferation by increasing the expression of two proliferative markers, known as cyclinE and proliferating cell nuclear antigen (PCNA) [79]. Airway epithelial cell dysfunction in asthma induces EMT through TGF-β signaling via noncanonical pathways (e.g., Wnt/β-catenin, JNK, Ras small GTPases, PI3K/Akt, p38) and canonical/Smad-dependent pathways (e.g., c-Jun/junB) activation [91]. Nicotine stimulates TGFβ1-induced EMT in hASM cells [89]. TGFβ1 stimulation causes a decrease in E-cadherin and an increase in MMPs, snail, twist, fibronectin, α-smooth muscle actin (α-SMA) and collagen 1 expression in airway epithelial cells [91]. TGFβ is increased by the existence of proinflammatory TNF-α/IL-1, which triggers the innate immune response in asthmatics through noncanonical and NF-κB signaling pathway activation [91]. It has been recently reported that miR-206 down-regulation mediates TGF-β1-induced cyclinD1 and HDAC4 up-regulation via activation of Smad2/3, resulting in significantly triggered hASM cell proliferation. However, activation of AMPK suppresses TGF-β1-induced hASM cell proliferation and miR-206 inhibition by downregulating HDAC4 and consequently decreased cyclinD1 [92]. It is well recognized that HIF-1α-induced EMT upregulates in bronchial epithelial cells [93]. Expression of HIF-1α and VEGF in hASM cells is upregulated in asthmatics compared to non-asthmatic patients [94], and that NF-κB signaling may be involved in IL-1β-induced HIF-1α activation through the canonical pathway of NF-κB [95].

CSE stimulates p120-mediated NF-κB activation in airway epithelial inflammation, which is dependent on the RhoA/ROCK signaling pathway [96]. Activation of the RhoA/ROCK signaling pathway increases airway inflammation in asthmatic patients. Upon activation by G protein-coupled receptor (GPCR) agonist, guanine nucleotide exchange factors (GEFs) enhance the active state by converting GDP-RhoA to GTP-RhoA. GTP-RhoA binds to Rho-kinase and causes contraction of airway smooth muscle. TNF-α, IL-13, IL-4, IL-17A and CCL2 are found to increase RhoA mRNA expression via NF-κβ and STAT6 signaling pathways activation, leading to airway hyperreactivity in asthma. Activation of TGF-β1 also has a key role in hASM contraction by increasing RhoA/ROCK-medicated phosphorylation of myosin phosphatase targeting subunit 1 (MYPT1) and myosin light chain (MLC) through PI3K signaling pathway activation [97].

Exposure to the nicotine component of CSE increases the expression of cleaved-caspase (C-caspase-3), cleaved-PARP (C-PARP) and Bax in human bronchial epithelial cells [98]. In bronchial mucous cells, the expression level of BAX mRNA was reduced in asthmatic non-smokers compared to non-asthmatics [99]. CSE induces PARP activation and DNA damage in hASM cells but did not induce apoptosis, cleave PARP or caspase 3 [100]. PARP activation may influence the pro-inflammatory cytokine/chemokine production in asthma, which is dependent on NF-κB signaling pathway activation [101]. Furthermore, CSE induces apoptosis in asthmatics in response to apoptosis-inducing factor, which has a key role in ROS-mediated cell-death and DNA damage [102].

Increasing iNOS and arginase I expression in airway epithelial cells of asthmatic patients after nicotine stimulation can result in nitric oxide (NO) production [103], which contributes to the pathological condition to increase the oxidative stress pathway and stimulate T-lymphocytes and eosinophil chemotaxis to the lung, leading to airway remodeling and inflammation [104].

Taken together, nicotine may contribute significantly to asthma by inducing cell proliferation, angiogenesis, inflammation and apoptosis of hASM/HBSMCs. These effects may occur via activation of signaling pathways mediated through the α7nAChR, including the PI3K/Akt and ERK/MAPK, as well as through canonical/Smad-dependent and noncanonical signaling pathway activation.

## 6. Therapeutic Role of *Scutellaria baicalensis* and Their Flavone Compounds in Nicotine-Induced Asthma

Few in vitro studies so far have tested the anti-allergic activity of flavone compounds in *S. baicalensis*. Some of these compounds are proven to be effective when tested on normal/allergic bronchial airway epithelial cells. However, it is still unknown how these compounds exert their therapeutic effects in nicotine-induced asthmatic bronchial airway epithelial cells. A study of normal bronchial epithelial (BEAS-2B) cells indicates that baicalein inhibits TNF-α-induced p-IκBα protein levels at a concentration of 2.5 μM/mL through inhibiting the NF-κB p65 signaling pathway [105]. In a study conducted by Dong et al. [106], human airway epithelial cells HBE16 were treated with 10 μg/mL lipopolysaccharide (LPS) to study the anti-inflammatory effect of baicalin on LPS-induced HBE16 cells. The results showed that baicalin inhibits p-IKKα/β and activates IκBα to suppress the LPS-induced NF-κB signaling pathway activation via binding TLRs, leading to inhibited TNF-α, IL-8 and IL-6 expression at the mRNA level.

The efficacy of flavone compounds in *S. baicalensis* to treat asthma is not yet confirmed. Thus, understanding conditions associated with bronchial airway wall remodeling in asthma might provide an opportunity to explore the therapeutic potential of flavone compounds in asthma. It is well known that bronchial fibroblasts and myofibroblasts are implicated in subepithelial fibrosis, a characteristic feature of bronchial airway remodeling in asthma [107]. In allergic asthma, the subepithelial fibrosis in lamina reticularis is caused by exaggerated deposition of extracellular matrix (ECM) protein components (e.g., fibronectin and collagen I, III and V) and α-SMA, which are activated by myofibroblasts [108]. Phenotypic fibroblast to myofibroblast transition in asthma is a process characterized by an increased number of myofibroblasts in fibrotic lung tissue as a result of EMT, and activated by several factors, including TGF-β, interleukins (e.g., TNF-α, IL-4, IL-5, IL-11, IL-13) and chemokines (e.g., periostin, osteopontin, eotaxin), which contribute to bronchial remodeling [108]. Asthmatic bronchial fibroblasts have been shown to be involved in NSCLC cell development. A previous study indicates that bronchial fibroblasts derived from asthmatic patients activate the colonization of lung tumors by increasing A549 cell motility and upregulating Snail-1/connexin43 (Cx43) expression in A549 cells [109].

Idiopathic pulmonary fibrosis (IPF) is considered a contributing factor to lung cancer, which shares characteristics with asthma such as inflammation and fibroblast proliferation that may link both diseases to the development of NSCLC. IPF, a chronic and progressive subtype of fibrosing interstitial lung disease (ILD) [110], increases the risk of NSCLC development and in smoking-related subtype SCC in particular [111,112], as a result of aberrant fibroblast proliferation, myofibroblast activation, oxidative stress, growth factor expression alterations and myofibroblast/mesenchymal transition [112]. In IPF lungs, it has been shown that TGF-β, VEGF, FGF, cytokines and chemokines (e.g., IL-13, CCL2), ROS, Wnt/β-catenin pathway, PI3K/AKT/mTOR pathway, Notch signaling pathway, ECM protein components, pulmonary arterial endothelial cell to mesenchymal transition (EnMT) and α-SMA are hypothesized as potential sources/mechanisms of myofibroblast activation, which contribute to lung fibrosis and lead to aberrant fibroblast proliferation and apoptosis of NSCLC cells [112]. Given these findings, this review hypothesizes that asthmatic bronchial airway epithelial cells may display a similar characteristic profile in cell surface markers with NSCLC cells, where studies are needed to further investigate possible mechanisms. Flavone compounds in *S. baicalensis* efficacy were confirmed by in vitro studies to exert a protective effect against nicotine-induced NSCLC, which may be due to the presence of C-6 and C-8 hydroxyl/methoxy groups in these flavones at the A-ring [39]. Therefore, this prompts us to hypothesize that these compounds may be involved in nicotine-induced asthma treatment. Table 3 shows the potential therapeutic activity of flavone compounds in nicotine-induced asthma. Future directions for exploring possible mechanisms underlying the therapeutic effects of these compounds in nicotine-induced asthma are warranted.

## 7. Conclusions

*S. baicalensis* and its flavone compounds exert therapeutic effects in nicotine-induced NSCLC. The therapeutic effect of *S. baicalensis* and its compounds against NSCLC cells induced by nicotine as an activator of α7nAChR are attributed to its ability to inhibit proliferation, invasion, migration, metastasis and angiogenesis, as well as inducing apoptosis, cell cycle arrest and autophagy via inhibiting signaling pathways involved in NSCLC development. Thus, inhibition of α7nAChR and its downstream signaling pathways by flavones compounds could be a potential target for drug development of nicotine-induced NSCLC cells and NSCLC treatment in smokers. The combination of flavone compounds with chemotherapeutic agents such as cisplatin that could modulate NSCLC-related signaling pathways is a potential strategy to increase the agent anti-NSCLC activity. Therefore, flavones compounds alone or in combination with chemotherapeutics could be approved anti-NSCLC medicinal drugs in smokers.

The mechanisms by which flavone compounds in *S. baicalensis* exhibit anti-asthma activity for use as medicinal drugs in nicotine-induced asthma remain unclear. Nicotine may contribute to asthma by inducing cell proliferation, angiogenesis, inflammation and apoptosis of hASM/HBSMCs through activation of cellular signaling pathways. Flavone compounds in *S. baicalensis* may have therapeutic potential in nicotine-induced asthma. The use of these compounds is not part of standard treatment of NSCLC and asthma. Integrative medicine is being part of the treatment of these diseases during last years as clinical trials with promising results. Further mechanistic studies to explore the link between asthmatic bronchial fibroblasts and NSCLC development are needed to ascertain the role of flavone compounds as potentially therapeutic in nicotine-induced asthma. Further studies are also needed to investigate the therapeutic efficacy and safety of *S. baicalensis* and its compounds for the treatment of smoker patients with NSCLC and asthma.

## Figures and Tables

**Table 1:** Major functions of nicotine/α7nAChR in NSCLC development.

Functions	α7nAChR-Associated Signalling Pathways in NSCLC
Cell proliferation	α7nAChR enhances cell proliferation by activating retinoblastoma tumor suppressor protein-proto-oncogene, serine/threonine kinase (Rb-RAF-1) and Src pathways mediated by protein β-arrestin. α7nAChR causes activation of phosphatidylinositol-3 kinase/Akt (PI3K/Akt), Sp1 transcription factor/GATA binding protein (Sp1/GATA1) and the mammalian target of rapamycin (mTOR) signalling pathways, which enhance proliferation of NSCLC cells. α7nAChR activates signalling pathways of PI3K/Akt/Src, leading to upregulation of cyclinD1 expression in NSCLC cells. α7nAChR promotes cell proliferation by the activation of nicotine-induced vimentin and fibronectin expression through the Raf-1/extracellular signal-regulated kinase/mitogen-activated protein kinase (Raf-1/ERK/MAPK) signaling pathway.
Metastasis	α7nAChR stimulates migration and invasion of NSCLC cells by activating the ERK/MAPK, Src/protein kinase Cι/FAK (Src/PKCι/FAK), PI3K and Yes-associated protein-E2F transcription factors 1 (YAP-E2F1) signaling pathways.
Angiogenesis	Upregulation of α7nAChR in NSCLC cells promotes angiogenesis via activation of Ca^2+^ influx, which stimulates signaling pathways including VEGF-A, NF-κB, PI3K/AKT and FGFR2.
Anti-apoptosis	α7nAChR suppresses apoptosis in nicotine-induced NSCLC cells by activating the PI3K/Akt pathway, which inhibits proapoptotic Bcl-2-associated X protein (Bax) expression. Nicotine upregulates the protein expression of B-cell lymphoma-2 (Bcl-2) through α7nAChR-mediated activation of the Raf-1/MAPK signaling pathway, which leads to phosphorylation of transcription factor c-myc, resulting in significantly inhibited apoptosis in NSCLC cells.

**Table 2:** Anti-lung cancer activity of *S. baicalensis* and their flavone compounds in nicotine-induced NSCLC.

*S. baicalensis*/*S. radix*/Flavones	Concentration	NSCLC Cell Lines Target	Activity	Molecular Mechanism Target	Reference
*S. baicalensis*	250, 500 µg/mL	A549	Inhibition of cell motility/proliferation, induction of G1 phase arrest	cyclinD1, CDK4, MMP2 ↓	[51]
*S. radix*	750 µg/mL	H2087, H358	Induction of apoptosis and autophagy	caspase 3, PARP, LC3-II/LC3-I, AMPK ↑ mTOR ↓	[52]
*S. radix*/baicalein/wogonin	42.3 μg/mL	A549	Inhibition of cell proliferation/invasion, induction of S phase arrest	cyclinD1 ↓, P53 ↑	[53]
*S. baicalensis*/baicalein/baicalin/wogonin	29.8, 27.5, 16.7 μg/mL	SK-LU-1, A549, SK-MES-1	Induction of apoptosis and S phase arrest	Bax, P53 ↑ cyclinA ↓	[54]
*S. baicalensis*/baicalein/baicalin/wogonin	1, 10, 50 µM	A549, H1299	Anti-metastatic, anti-inflammatory, induction of apoptosis	Bax ↑ MMP2,MMP9, caspase-3, bcl-2/bax, bcl-2, NF-κB p65, TNF-α, IL-6, IκB-α ↓	[55]
*S. baicalensis*/baicalein/baicalin/wogonin	10, 40, 200 μM	A549, H1299	Inhibition of cell migration/invasion, Id1 inhibition, anti-angiogenesis	VEGF-A, N-cadherin, vimentin, a7nAChR, Akt/Src ↓ E-cadherin ↑	[41]
Baicalein	40 µmol/L	A549, H460	Inhibition of cell proliferation, induction of apoptosis	PI3K/Akt, miR-424-3p ↓	[56]
Baicalein	40 µmol/L	A549	Inhibition of cell motility/viability, anti-angiogenesis	PI3K, MMP2, MMP9 MMP14, VE-cadherin, RhoA/ROCK ↓	[57]
Baicalein	100, 200 µM	A549, H1299	Induction of autophagy	MAP4K3, mTOR ↓	[58]
Baicalein	10, 40 µmol/L	A549, H1299	Inhibition of cell invasion/metastasis	ezrin tension transduction, leader cells production, iNOS ↓	[59]
Baicalein	1, 10, 100 µM	A549, H460, SKMES1	Anti-angiogenesis	VEGF-A, FGFR-2, RB-1 ↓	[42]
Baicalein	80 µmol/L	A549, H1299	Inhibition of cell proliferation/invasion, Notch1 and hes-1 expression	cyclinD1, CDK1, N-cadherin, vimentin ↓ E-cadherin ↑	[60]
Baicalein	10 µM	A549	Anti-proliferative	N-cadherin, vimentin, Src/Id1 ↓ E-cadherin ↑	[43]
Baicalin	100 µM	A549, H2009	TRAIL-induced apoptosis	MAPK, ROS ↑	[61]
Baicalin	2, 4, 8 μg/mL	A549/DDP	Inhibition of cell proliferation/invasion	MARK2 mRNA, p-Akt ↓	[62]
Baicalin	25, 50, 100 µM	H441, H1975, H1299	Inhibition of cell proliferation/invasion	PBK/TOPK ↓	[63]
Baicalin	20, 40, 80 µmol/L	A549, H1299	Inhibition of cell proliferation/invasion induction of apoptosis	SIRT1/AMPK ↑ MMP2, MMP9 ↓	[64]
Wogonin	20 µM	A549	Induction of apoptosis, cisplatin-induced cell death	caspase 3, PARP, ROS **↑**	[65]
Wogonin	20 µM	A549	Inhibition of cell migration/metastasis, anti-inflammatory	(IL-6)-induced EMT, N-cadherin, vimentin, Snail, Twist, STAT3 ↓ E-cadherin ↑	[66]
Wogonin	15 μg/mL	A549	Inhibition of cell proliferation, glucose metabolism alteration	LDH, ATP synthesis ↓	[67]
Wogonin	50 µM	A549, A427	Induction of apoptosis and autophagy	caspases 8/9/3,ROS, LC3II **↑**	[68]
Wogonin	35 μg/mL	A549	Inhibition of cell viability, induction of apoptosis	HDAC1/2, Skp2, Fbw7, GSK3b ↓	[69]
Wogonin	10 µM	A549	Cell cycle arrest, senescence, apoptosis	APAF1, Bax, p21, PML, GADD45A, YPEL3 **↑** SGK1 ↓	[70]
Wogonoside	80 μM	A549	Induction of apoptosis, cell cycle arrest	caspases 3/9, Bax, mitochondrial cytochrome c, AMPK ↑ Bcl-2, mTOR ↓	[71]
Wogonin	60, 100, 200 μM	DMEM	Anti-proliferative	caspases 3/9 **↑**	[72]
Oroxylin A	40 μM	H460	Anti-inflammatory	T_regs_, TGFβ, Smad3, ERK1/2, JNK, P38, NF-κB/p65, IKKα, IKKβ ↓	[73]
Oroxylin A	16 μM	A549,95-D	Anti-invasive/migration	Snail, ERK/GSK-3β, CD44v6, MMP-9, vimentin ↓ E-cadherin **↑**	[74]
Oroxylin A	120 μM	A549	Anti-invasion, glucose metabolism alteration	Src/PI3K/AKT, ATP synthesis, lactic acid formation, HK II ↓	[75]
Oroxylin A	50 μmol/L	H460	Suppression of XPC	HIF-1α ↓	[76]

(↓) decrease, (↑) increase.

**Table 3:** Therapeutic potential of flavone compounds from *S. baicalensis* in nicotine-induced asthma.

Activity	Mechanism of Action
Anti-inflammatory	Downregulate expression of pro-inflammatory cytokines (IL-4, IL-6, IL-8, IL-17A, TNF-α/IL-1) and chemokines (CXCR1, CXCR2) in hASM cells through inhibiting TGF-β1-induced EMT, p-Smad3, ERK1/2 and p38 MAPK, and noncanonical and NF-κB signaling pathways Suppress expression of TNF-α, IL-13, IL-4, IL-17A and CCL2 via inhibiting NF-κB, STAT6, PI3K and RhoA/ROCK signaling pathways activation, which may lead to downregulation of RhoA mRNA expression
Anti-proliferation	Inhibit α7nAChR-mediated PI3K/Akt signaling pathway activation and downregulate TRPC6 mRNA and protein expression, which may lead to decreased ROCE, SOCE and basal [Ca^2+^]_i_ levels in HBSMCs Suppress Orai1, CD38, STIM1 expression and TRPC3-mediated SOCE in hASM cells Downregulate TGF-β1-induced cyclinD1, cyclinE, PCNA and HDAC4 and up-regulate miR-206 expression through inhibiting Smad2/3 signaling pathway activation
Anti-angiogenesis	Downregulate expression of VEGF and IL-1β-induced HIF-1α activation in hASM cells via suppressing NF-κB signaling pathway Suppress MMPs, snail, twist and fibronectin, and up-regulate E-cadherin expression through noncanonical signaling pathway inactivation
Anti-apoptosis	Inhibit NF-κB signaling pathway activation and ROS production, which may lead to decreased PARP and BAX expression in hASM/HBSMCs

## Data Availability

Not applicable.
